# Bone Morphogenetic Protein-2 in Development and Bone Homeostasis

**DOI:** 10.3390/jdb8030019

**Published:** 2020-09-13

**Authors:** Daniel Halloran, Hilary W. Durbano, Anja Nohe

**Affiliations:** Department of Biological Sciences, University of Delaware, Newark, DE 19716, USA; dhallor@udel.edu (D.H.); weidnerh@udel.edu (H.W.D.)

**Keywords:** development, BMP-2, CK2, CK2.3, osteoporosis, osteoblasts, osteoclasts, Smad1/5/8

## Abstract

Bone morphogenetic proteins (BMPs) are multi-functional growth factors belonging to the Transforming Growth Factor-Beta (TGF-β) superfamily. These proteins are essential to many developmental processes, including cardiogenesis, neurogenesis, and osteogenesis. Specifically, within the BMP family, Bone Morphogenetic Protein-2 (BMP-2) was the first BMP to be characterized and has been well-studied. BMP-2 has important roles during embryonic development, as well as bone remodeling and homeostasis in adulthood. Some of its specific functions include digit formation and activating osteogenic genes, such as *Runt-Related Transcription Factor 2* (*RUNX2*). Because of its diverse functions and osteogenic potential, the Food and Drug Administration (FDA) approved usage of recombinant human BMP-2 (rhBMP-2) during spinal fusion surgery, tibial shaft repair, and maxillary sinus reconstructive surgery. However, shortly after initial injections of rhBMP-2, several adverse complications were reported, and alternative therapeutics have been developed to limit these side-effects. As the clinical application of BMP-2 is largely implicated in bone, we focus primarily on its role in bone. However, we also describe briefly the role of BMP-2 in development. We then focus on the structure of BMP-2, its activation and regulation signaling pathways, BMP-2 clinical applications, and limitations of using BMP-2 as a therapeutic. Further, this review explores other potential treatments that may be useful in treating bone disorders.

## 1. Introduction

Bone Morphogenetic Proteins (BMPs) are multi-functional growth factors belonging to the Transforming Growth Factor-Beta (TGF-β) superfamily, which is also shared by Growth Differentiation Factors (GDFs), Glial-derived Neurotrophic Factors (GDNFs), Lefty, Inhibins, Activins, Nodal, and Mülllerian Inhibiting Substance (MIS) [1,2,3,4,5]. Currently, over 20 BMPs have been identified, making these proteins the largest subgroup within the TGF-β superfamily. BMPs have crucial functions in developmental processes, such as cardiogenesis, digit apoptosis, somite formation, neurogenesis, eye formation, and musculoskeletal development [6,7,8,9,10,11,12,13]. Additionally, BMPs are expressed throughout adulthood, contributing to osteogenesis, adipogenesis, chondrogenesis, programmed cell death, cardiac differentiation, and nervous system maintenance [14,15,16,17,18,19,20,21,22,23,24,25,26]. The first BMP was identified in bone in 1965 by Marshall Urist and named BMP-2 [27]. Since then, BMP-2 has been well-characterized and many of its functions in bone and throughout the body have been elucidated. Because BMP-2 has been extensively studied and characterized, especially during development and in bone, this review will focus on BMP-2 in development and osteogenesis. 

After identifying BMP-2, subsequent studies focused on BMP-2 and demonstrated its critical role during and after development. In early development, BMP-2 is critical for successful digit formation, cardiogenesis, neuronal growth, and many other processes [6,20,21,22,25,28,29,30]. It is continuously expressed into adulthood, where it induces processes such as intramembranous and endochondral ossification [2,27,31,32]. Additionally, BMP-2 is expressed in many cells throughout the body, such as osteocytes and osteoblasts [33]. Further elucidating the mechanism of BMP-2, Knockouts (KOs) and conditional KOs of BMP-2 in mice led to lethality or underdeveloped bones with reduced radial bone thickness, strength, and increased risk of spontaneous fractures; further, these KOs presented heart deficiencies, chondrocyte abnormalities, and reduced vasculature [14,32,34,35,36,37].

BMP-2 has additional essential functions in the musculoskeletal system. Bone itself is a very dynamic and complex organ. The human skeleton provides protection of vital organs, structure, locomotion through anchoring of muscles, and mineral homeostasis [36]. Five major types of bone comprise the skeleton and dependent on their size and shape, these bones exert specific functions. Classified based on their shape, they include short (carpals), flat (ribs), long (femur), irregular (vertebrae), and sesamoid bones (patella). Each of these bone types have a specific function based upon their overall shape and morphology. In addition, each bone has four major layers of bone tissue: the periosteum is the outermost protective layer; cortical or compact bone is the outer dense shell located underneath the periosteum; cancellous or trabecular bone, also known as spongy bone, is located on the inside of the cortical shell; and finally there is the marrow cavity where important or critical stem cells, such as mesenchymal stem cells (MSCs) and hematopoietic stem cells (HSCs), are located [38,39]. The marrow cavity is vital, because as adults age, the locations of stem cells diminish, leading to complete reliance on the bone marrow cavities to promote differentiation of these pivotal cells. HSCs and MSCs differentiate into bone cells that are responsible for resorbing back old or damaged bone and building new bone, however, they are also responsible for differentiating cells into adipocytes or fat cells [40,41]. While adipocytes are important for bone growth and remodeling, too many fat cells within the bone are detrimental as this causes increased porosity and decreased structural support. This leads to an increase risk of fractures and breaks within the bone. Therefore, the correct balance between bone resorption, bone formation, and fat cell formation is critical for the bone remodeling process [42].

**Figure 1:**
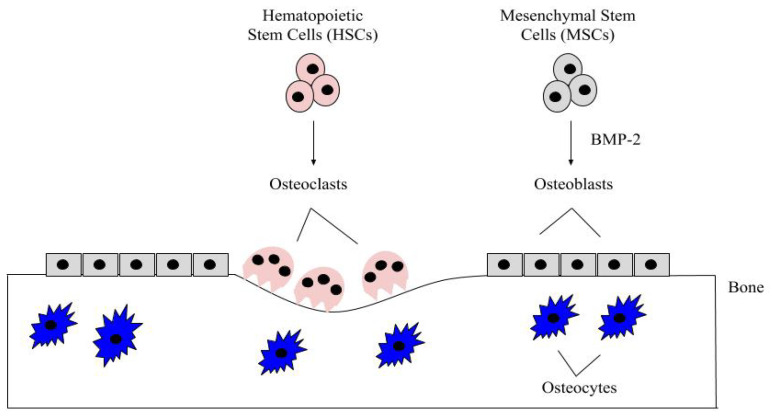
The bone microenvironment. Healthy bone function and renewal is controlled by the activity of osteoblasts and osteoclasts. Osteoblasts derive from MSCs, which commit to the osteoblast lineage after exposure to BMP-2. BMP-2 is secreted into the bone matrix or bloodstream by pre-existing osteoblasts, osteocytes, and endothelial cells, where it can bind to bone morphogenetic protein receptors (BMPRs) on MSCs. After MSCs differentiate into osteoblasts, these cells secrete the organic matrix of the bone. Some eventually become embedded within the bone as osteocytes, which provide further structure. HSCs differentiate into osteoclasts after being stimulated with factors, such as RANK-L and NF-kB. The osteoclasts are multinucleated and resorb the bone matrix, releasing contents (i.e., BMP-2 and calcium) to be recycled throughout the body.

**Figure 2:**
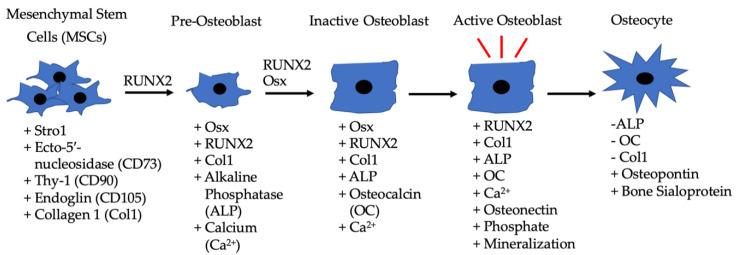
Differentiation of MSCs to osteoblasts driven by BMP-2, along with the other listed factors. MSCs are located in bone marrow and differentiate into pre-osteoblasts when exposed to RUNX2 and BMP-2. The pre-osteoblasts differentiate into inactive osteoblasts in the presence of RUNX2, BMP-2, and Osx. Inactive osteoblasts become active osteoblasts in bone microenvironments that require rebuilding, and BMP-2 can also assist in this process. Active osteoblasts can then become embedded in bone and function as osteocytes.

Bone remodeling is a critical process and it occurs continuously in the adult skeleton. In fact, approximately 10% of human bone is remodeled every year [43]. The bone is remodeled by resorption of old bone by osteoclasts and formation of new bone by osteoblasts (Figure 1). Osteoclasts in this microenvironment function as innate immune cells, initiating inflammatory responses and resorbing old or damaged bone [44,45]. When new bone needs to be formed, MSCs can differentiate into osteoblasts, and these osteoblasts can become embedded in the bone as osteocytes, which provide additional bone support and structure [41]. The main factor that differentiates MSCs into osteoblasts is BMP-2, which is released within the bone matrix or serum during osteoclast-driven bone resorption (Figure 2). To differentiate MSCs into osteoblasts, BMP-2 binds to type I and type II serine/threonine kinase receptors on target cells, activating Smad (canonical) and non-Smad (non-canonical) signaling pathways, which ultimately activates osteogenic genes such as *Runt-Related Transcription Factor 2* (*RUNX2*) and *Osterix* (*Osx*) [2]. Furthermore, BMP-2 is also important in stimulating osteoclastogenesis by directly differentiating osteoclast precursor cells into osteoclasts, and antagonizing BMP-2 led to the downregulation of this process [46,47]. As BMP-2 is important for MSC differentiation, osteogenesis, and osteoclastogenesis, recombinant human BMP-2 (rhBMP-2) was approved by the Food and Drug Administration (FDA) and is administered after lumbar spinal fusions in tapered or cylindrical interbody cages [48,49,50,51]. Although BMP-2 promotes fracture healing, several post-surgical complications arose, including ectopic bone formation, radiculitis, vertebral osteolysis, increased microfracture occurrence, low bone healing efficacy, and hematoma formation [48,52,53,54,55,56,57]. The molecular mechanisms implicating the ectopic bone induction process is not well understood; however, it is believed to follow a non-physiological pathway. There have been promising strides in this particular area of research, as Hashimoto and colleagues developed a novel imaging method that can help both visualize and determine ectopic bone formation pathways. This discovery will help to facilitate and direct BMP-2 induced ectopic bone formation in concert with other molecules, like PTH or IL-17; however, more research is needed [58]. Therefore, a new treatment for bone disorders must be developed. In this review paper, we will describe the structure of BMP-2, its role in development, and its receptors, along with how it is regulated by agonists and antagonists to activate various signaling pathways. Additionally, we will describe the regulation of BMP-signaling pathways and how this regulation led to current and alternative therapeutics for bone disorders. As the FDA has approved BMP-2 for use in skeletal disorders and fractures, bone and bone disorders will be the primary focus of this review. 

## 2. BMP-2 and Development

BMP-2 has many roles during development. From the earliest stages of embryogenesis, BMP-2 regulates the formation of the dorsal/ventral (D/V) and anterior/posterior (A/P) axes [59,60,61,62]. While regulating formation of the axes, BMP-2 is also involved in somite formation and somatic chondrogenesis, especially in the vertebral and axial skeleton [63,64,65]. Further, BMP-2 is crucial in nearly every stage of neural development and is essential for closure of the neural tube [11,12,22,66]. BMP-2 is also involved in the development of the optical system. Specifically, it assists in the remodeling of the sclera as well as contributing to the formation of the retinal system [13,67,68]. However, overexpression of BMP-2 can lead to early myopia, and thus, its activity must be tightly regulated during embryogenesis and throughout development [67,68,69]. In addition to cell patterning, neurogenesis, and eye development, BMP-2 is involved in digit formation. BMP-2 assists in programmed cell death to initiate apoptosis in distal limbs, allowing for the formation of digits; lack of BMP-2 leads to the malformation of digits [7,20,28,70]. Simultaneously, BMP-2 is involved in formation of the mesoderm and in cardiogenesis [34]. BMP-2 has a prominent role during epithelial-mesenchymal transition (EMT) and formation of myocardial cells to ensure proper heart development [6,23,71]. BMP-2 KOs in mice led to malformation of the heart, heart valves, and irregular myocardial patterning that resulted in lethality during early embryonic stages [23,24,26,34,35]. Further, BMP-2-deficient mice exhibited malformation of both the chorion and amnion, suggesting BMP-2’s diverse role in many cardiac developmental processes [35,72,73,74]. While assisting in the cardiogenesis, BMP-2 is also involved in the formation of the pulmonary system. For example, when the lung begins to develop in early embryonic stages, BMP-2 activates BMP-signaling pathways and stimulates formation of alveolar cells and regulates pulmonary remodeling [75,76,77]. BMP-2 also regulates pulmonary specification and branching to increase alveolar surface area [76,77]. Lastly, BMP-2 is required for proper osteogenesis, chondrogenesis, and adipogenesis during development [2,16,18,78]. BMP-2 is the ligand needed to activate the Smad and Non-Smad pathways that lead to bone, cartilage, and fat development; KOs or under-expression of BMP-2 result in the inability of these cells and tissues to form [18,32,79,80]. Thus, BMP-2 has a prominent role in many stages of development and demonstrates its multi-functionality. 

### 2.1. BMP-2 Receptors and Expression

Over the past decades, researchers discovered several mutations within the *BMP-2* gene, revealing the importance of BMP-2 in regulating bone homeostasis and survivability. For example, mutations in the *BMP-2* gene can cause altered signaling leading to brachydactyly and lethality, as downstream effector proteins and signaling pathways are not effectively activated [34,81,82,83]. In order for BMP-2 to elicit its many cellular responses, it must bind to specific receptors and activate a signaling cascade. However, before investigating the functional role of BMP-2, we first must investigate how BMP-2 becomes functional (or how it is transcribed and translated). Once the *BMP-2* gene is transcribed and translated, BMP-2 is not yet functional. To become active, the newly formed preproprotein undergoes proteolytic cleavage by proprotein convertase subtilisin/kexin type 5 (PCSK5) at the C-terminus [84]. The functionally cleaved 115 amino acids (AA) BMP-2 protein is secreted from the cell to serve as an autocrine or paracrine factor, binding to receptors on both osteoblasts and osteoclasts [46,47,85]. Once released into the bone matrix or blood, BMP-2 can be processed further by proteases. Specifically, these proteases located in the serum are able to cleave BMP-2, 4, and 7, which can de-differentiate newt muscle cells and allow them to re-enter the cell cycle. Here, the de-differentiated cells are able to commit to other cell fates, dependent on which factors they are exposed to [86,87]. Further studies elucidating this mechanism in other animals should be explored.

BMP-2 binding to BMP type I and type II serine/threonine kinase receptors leads to activation of several downstream signaling pathways along with upregulation of the *BMP-2* gene. Cells can express various types of receptors located on the cell surface. BMP-2 can bind to BMP receptor type Ia (BMPRIa), BMP receptor type Ib (BMPRIb), and activin receptor type Ia [88,89]. BMPRIa is located on most cell surfaces while BMPRIb is less common [90,91,92]. BMP-2 preferentially binds to preformed BMPRII-BMPRIa/b complexes or binds to BMPRIa specifically at its beta4beta5 loop, which can then oligomerize with BMPRII [93,94]. Additionally, BMPRII can also oligomerize with BMPRIa/b alone when BMP-2 binds, which causes the type I receptors to oligomerize with the type II receptors and activates different signaling pathways [95]. BMPRs are known to be expressed on both osteoblasts and osteoclasts, which is critical for bone remodeling and homeostasis. On these cells, BMP receptor signaling is also regulated by the localization of receptors in specific membrane domains. Receptors can be localized in caveolae, clathrin coated pits (CCPs), and lipid rafts located on the plasma membrane [96,97,98,99,100]. Their localization can determine which signaling pathways are activated [96,97,99,101]. Because BMPRs regulate many pathways, mutations can be detrimental. For example, in early stages of development, most gene mutations within the *BMPRIa* gene result in a shorter protein sequence, which leads to inadequate BMP signaling as the specific ligands are unable to bind to BMPRs [102,103,104,105,106,107]. *BMPRIa* has about 60 known mutations that cause juvenile polyposis syndrome, which can lead to unregulated cell growth and causes ectopic polyp formation [102,103,104,105,106,107]. Additionally, BMP-2 can bind to three type II receptors, including BMP receptor type II (BMPRII), activin receptor type IIa (ActRIIa), and activin receptor type IIb [1,108]. BMP-2 binds to BMPRIa with the highest affinity [94,109].

Once BMP-2 binds to the BMPRs, phosphorylation of BMPRIa by BMPRII leads to adipogenesis, chondrogenesis, and osteogenesis, whereas phosphorylation of BMPRIb leads to apoptosis and cell death [28,34,95,110,111,112]. Further, different patterns of receptor oligomerization determine the downstream pathways BMP-2 activates. For example, Smad signaling is activated when BMP-2 binds to preformed heteromeric complexes, whereas Non-Smad signaling (such as ERK) is activated when BMP-2 binds to BMPRIa and BMPRII is recruited after [18,19,109]. Therefore, localization and oligomerization of BMPRs will determine the signaling response after BMP-2 binds. [16,17,113,114,115]. 

In addition to BMPRs, there are co-receptors, such as BMP and activin membrane-bound inhibitor (BAMBI), Dragon, endoglin, and betaglycan that can be associated with BMPRIa, which can enhance or inhibit BMP-signaling [112]. For example, BAMBI is a pseudoreceptor localized near BMPRs. When BMP-2 binds, the BMPRs activate BAMBI, and BAMBI decreases the BMP-signaling response as a negative regulator [116,117,118]. Additionally, DRAGON enhances BMP-signaling during development, especially in the nervous system [119,120,121]. Endoglin, a type 1 transmembrane glycoprotein, is a co-receptor of BMPRII and is crucial for cardiogenesis and angiogenesis, along with influencing Non-Smad signaling by affecting cell growth and adhesion [122,123,124]. Betaglycan is a commonly expressed proteoglycan that serves as a co-receptor to BMPRII. One of its functions is to negatively regulate BMP-signaling by activating inhibin, which then associates with the receptor and prevents BMP-2 binding [125,126,127,128]. Along with these co-receptors and associated proteins, previous studies have demonstrated a Heparin-binding site on BMP-2 that regulates its activity [129,130,131,132]. This Heparin-binding site regulates BMP-2’s activity as when it travels through the ECM of cells, it binds to ECM proteins, such as fibronectin and tenascin C [133,134,135]. This binding limits the migration and movement patterns of BMP-2, limiting its bioavailability and activity throughout the body, which may be useful when sustaining delivery of rhBMP-2 in clinical applications [136,137]. This is further demonstrated as when this binding site is mutated, BMP-2 activity increased [129,138]. This extensive regulation of BMP-2 is a combination of negative feedback, co-receptors/proteins, and binding domains.

Further, studies have demonstrated that BMP-2 is expressed in a variety of cells. By being expressed largely throughout the body, BMP-2 is able to effectively function and activate the many signaling pathways listed above. For example, BMP-2 is expressed in many tissues, including the liver and the lungs, as well as in bone (primarily in osteoblasts and osteocytes) [37,139]. Additionally, BMP-2 can be paracrine or autocrine, acting as a local or systemic factor to initiate cell-to-cell responses or travelling through the serum to target cells [140,141,142]. Further, in the absence of BMP-4, BMP-2 has been shown to compensate for its functions, especially in chondrocytes, bone, and during development [32,143]. These expression patterns are vital to ensure proper maintenance of alveolar tissue, hepatocytes, developmental processes, and bone homeostasis. 

### 2.2. BMP-2 Structure and Receptor Binding

BMP-2 was first discovered in 1965 due to its potent osteo and chondro-inductive abilities; however, the structure of BMP-2 was not crystallized until 1999. After BMP-2 was synthesized as a 453 residue proprotein, it became glycosylated and further proteolytically cleaved and dimerized. This yields the final, mature disulfide linked homodimer. Each respective monomer is 114 residues in length. A 3D rendering of the dimer’s crystallized structure revealed that the biologically active form of this protein has the dimensions of 70 Ǻ × 35 Ǻ × 30 Ǻ, where the center of the monomer is 10 Ǻ thick. Each individual monomer contains a cystine-knot formed through six cysteine residues creating three intrachain disulfide bridges. This structure is critical for BMP-2, as this provides stability that would otherwise be lacking due to the lack of a hydrophobic core. However, this is shortly resolved once the two monomers form the dimer, further stabilizing the structure of BMP-2, as well as creating extra stability through the creation of a hydrophobic core between the two monomers. The folding topography of BMP-2 features the key components of other TGF-β superfamily proteins. Briefly, they consist of two separated antiparallel β-sheets, which is made up of nine β strands. The strands do not form four antiparallel β sheets because they too far apart to participate in hydrogen bonding. The second sheet adopts a twisted crossover confirmation. There is also a four-turn α-helix which is located perpendicular to the β strands [144]. 

BMP-2, like BMP-4, can exist in a soluble form so that it can be easily transported. When BMP-2 is in its soluble form, it binds to a lower affinity to its type II receptor, BMPRII [145,146]. However, in most cases, BMP-2 preferentially binds to the type I receptors, most notably BMPRIa [21,147]. In order to determine exactly how BMP-2 and BMPRIa binding is achieved and facilitated, the bound crystal structure needed to be resolved. Once crystallized, it was found that BMP-2 bound to BMPRIa through the figure-helix groove of the BMP-2 dimer. It is bound in such a way that both monomers of BMP-2 come into contact with the receptor and the C-terminus of the receptor chains are found 65 Ǻ apart. The general structure of BMPRIa can be likened to a left hand where the thumb is the helix backbone, the three extended middle fingers are the central beta sheet, and the small or pinky finger is slightly bent, indicating a loop between the β1–β2 sheets. In 2001, two binding epitopes on BMP-2 were discovered, known as the “wrist” and “knuckle” epitopes. The wrist epitope encompasses a larger area, correlating with high affinity to BMPRIa binding, while the knuckle epitope encompasses a smaller area that has low affinity for BMPRII binding. This is because binding residues located within the knuckle epitope are found in only one BMP-2 monomer, while binding residues in the wrist epitope are found in both monomers. Interestingly, the distances between the wrist and knuckle epitopes is 10–15 Ǻ, but the distances between the two receptors are much larger: 40–55 Ǻ. This distance between the two receptors helps to further stabilize the overall bound structure of BMP2 and its receptors [148]. 

There are several regulatory feedback mechanisms which help to control BMP-2 induced activity. One of these mechanisms is through BMP antagonists, like Noggin. It was discovered that the structure and complex of Noggin inhibited BMP signaling by directly binding and blocking critical epitopes on both the type I and type II receptors. Noggin is a twelve-membered cysteine knot protein; therefore, it can mimic those wrist and knuckle epitopes that BMP-2 contains, in order to facilitate competitive binding to the BMP receptors, thus inhibiting BMP-2 induced signaling [149]. This means both the location and proximity of the receptors is critical for proper BMP-2 binding and subsequent pathway activation [148]. 

### 2.3. BMP-2 Signaling Pathways

**Figure 3:**
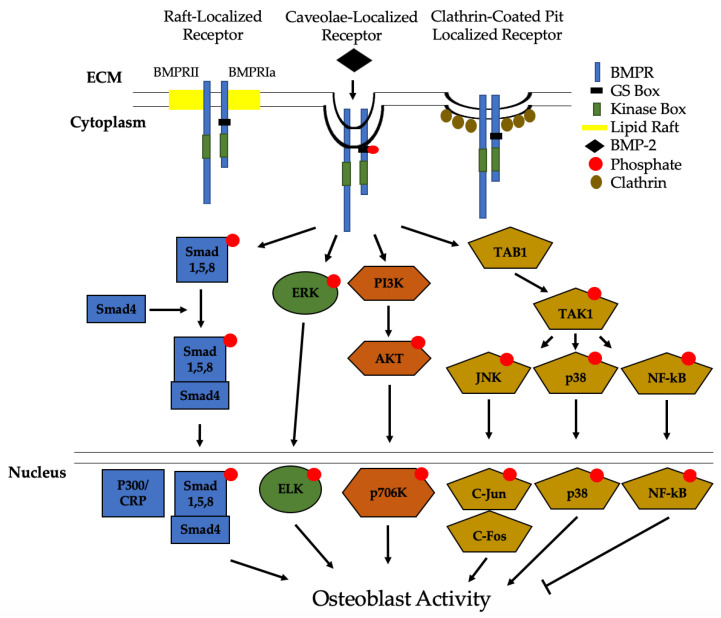
Bone Morphogenic Protein 2 (BMP-2) activation of signaling pathways. Once BMP-2 binds to the BMPRs located in lipid rafts, caveolae, and clathrin coated pits (CCPs), constitutively active BMPRII phosphorylates BMPRIa. This leads to downstream activation of the Smad pathway or the non-Smad pathways. In non-Smad signaling, the extracellular signal-related kinase (ERK), phosphatidylinositol 3-kinase (PI3K), and the transforming growth factor-β-activated kinase 1/binding protein 1 (TAB1/TAK1) pathways are activated. All of these pathways, except for NF-kB, lead to an increased differentiation of MSCs and osteoprogenitors into osteoblasts.

Many important signaling pathways for osteogenesis, cell survival, and apoptosis are activated by BMP-2 [20,22,28,41,150]. Once BMP-2 binds to BMPRs, it activates Smad and Non-Smad signaling pathways (Figure 3). The Smad pathway is activated when BMPRIa and BMPRIb phosphorylate downstream proteins, namely Smad1/5/8 [109]. The phosphorylated Smads recruit Smad4, and the complex translocates into the nucleus and acts as a transcription factor for genes, such as *RUNX2* and *Osx* [15,151]. Additionally, in specific circumstances, BMP-2 can also activate Smad2/3 signaling through BMPRIa. In fact, Smad2/3 was preferentially activated in embryonic and transformed cells, suggesting the promiscuity of BMP-2 to regulate developmental processes and cell division [152,153,154]. However, this process is not fully understood and BMP-2 may also exert this response in other cells. Thus, future studies should explore this area to delineate more of BMP-2’s actions and current information about the promiscuity of BMP-2 can be found in a review article by Nickel and Mueller [155]. 

Recently, we identified Casein Kinase 2 (CK2) as a key regulator of the BMP-signaling pathway [156]. Without the BMP-2 ligand present, CK2 is bound to BMPRIa, preventing the activation of downstream effector proteins. However, when BMP-2 is bound, CK2 is released and an upregulation of osteogenesis is observed [156,157,158]. In the non-Smad pathway, MAPK signaling activates extracellular signal-regulated kinase (ERK), phosphatidylinositol-2 kinase (PI3K), and the TAB1/TAK1 pathways [31,156,157,158,159]. Each of these signaling events, except for TAB1/TAK1 which activates NF-kB and p38, leads to differentiation of osteoblast precursors into osteoblasts. NF-kB has been shown to inhibit osteoblast function in osteoporotic mice models and requires further investigation [160,161]. Other pathways that can be activated by BMP-2 to differentiate myoblasts and other pre-osteoblasts into osteoblasts include EIF2AK3-EIF2A-ATF4 and RhoA/Rb [162,163,164]. 

As mentioned previously, BMPR localization on the plasma membrane determines endocytosis and which signaling pathways are activated. Previous studies first demonstrated that BMPRs were predominantly localized to CCPs [97]. However, subsequent research indicated that BMPRs also localize to caveolae, and that caveolae are essential regulators of Smad-signaling pathways [96]. Data demonstrated that BMP-2 preferentially binds to BMPRIa aggregates in caveolae with a higher force and frequency than CCPs to activate Smad signaling pathways [96,99,101,114,165]. Although BMPRs are primarily found in caveolae or CCPs, they can also localize on lipid rafts [97,98,114]. Additionally, BMP-2 has been shown to activate the Wnt signaling pathway; however, the proteins involved and whether BMP-2 induces the Wnt/β-Catenin pathway, or vice-versa, are still unknown [157,166,167]. 

### 2.4. Intracellular and Extracellular Regulation of BMP-2

Signaling cascades for any cellular pathway must be regulated, and BMP-2 signaling is no different, especially due to the multiple signaling pathways activated by BMP-2. Various proteins have been identified to regulate this response. The activity of BMP-2 is enhanced or inhibited intracellularly and extracellularly. For instance, the genes *Twisted gastrulation* (*Tsg*) and *Shrew* promote BMP-2 activity and enhance its activity [168,169,170]. Further, BMP-2 is regulated intracellularly and extracellularly by several secreted antagonists. Factors such as noggin, sclerostin (SOST), and follistatin directly bind to BMP-2 in the ECM to prevent its interaction with BMPRs, especially during development [171,172,173]. Specifically, other ECM proteins, including fibronectin, fibrinogen, and tenascin C, are able to bind to the Heparin domains of BMP-2, regulating its activity and migration [130,133,135,174,175,176]. Additionally, chordin is a protein secreted from bone cells in the spine that can directly bind to BMP-2 and prevent its interaction with BMPRs [170,171,177]. Intracellularly, inhibitory Smads (I-Smads) 6 and 7 directly regulate Smad signaling induced by BMP-2, usually by preventing downstream signaling cascades [178]. Despite these multiple regulations, abnormal bone and cartilage loss still occurs in osteoporotic and osteoarthritic patients. The underlying mechanisms are an active area of present research, but it is hypothesized that osteoporotic patient osteoblasts have irregular BMPRIa function, leading to dysregulation of BMPR trafficking and bone homeostasis [179]. The current identified agonists and antagonists of BMP-2 that may be involved are summarized in Table 1. Furthermore, we also include known associated proteins with BMPRs that limit the activity of BMP-2 in Table 2. 

### 2.5. Regulation of the BMP-2 Signaling Pathway by Casein Kinase 2 (CK2)

Recently, CK2 was not only identified as a key regulator of the BMP-2 pathway, but also as an inhibitor of this pathway. When inhibiting this pathway, CK2 is bound to three phosphorylation sites of BMPRIa, preventing the activation of downstream proteins [156]. To observe the interaction of BMPRIa and CK2, we constructed mutants of BMPRIa for each phosphorylation site at specific amino acids (AA 213–217; AA 324–238; AA 475–479) were constructed [157]. The BMPRIa mutants led to increased adipogenesis, osteogenesis, and chondrogenesis by preventing the binding of CK2 [158]. Additionally, to understand the relation between CK2 and BMPRIa, our lab deleted the *BMPRIa* gene in mice and surprisingly, this led to increased bone formation [180,181]. Next, the Nohe lab designed peptides mimicking the three phosphorylation sites for BMPRIa were constructed and named CK2.1, CK2.2, and CK2.3. These peptides were able to bind and prevent CK2 from interacting with BMPRIa, leading to increased adipogenesis, osteogenesis, and chondrogenesis, similar to the BMPRIa mutants [31,115,156,157,182,183]. Additionally, overexpression of a BMPRIa mutant (SLKD) that lacked a serine AA to prevent binding of CK2 led to increased mineralization via extracellular signal-related kinase/,itogen-activated protein kinase kinase (ERK/MEK) signaling, indicating that the BMP-2 signaling pathway is regulated by CK2 [157,158]. This suggests that exogenous BMP-2 is not needed to activate downstream pathways if CK2 inhibitors are present.

### 2.6. Endocytosis and Degradation of BMP-2 and BMPRs

As stated in previous sections, BMP-2-BMPR complexes can be endocytosed into cells via CCPs, caveolae, or lipid rafts. However, upon endocytosis, the complex continues as a signaling endosome, and is degraded (or recycled) to the plasma membrane [213]. First, to regulate the signaling activity of BMP-2 after endocytosis, this protein must be deactivated or degraded to prevent continuous expression. To suppress BMP-2 activity, data demonstrated that BMP-2 is ubiquitinated at several lysine residues, initiating its degradation [214]. Further, if this proteasomal-ubiquitination pathway is inhibited, BMP-2 activity is increased, and the protein is secreted more rapidly from cells [214,215]. However, the time-course of BMP-2 degradation and the mechanism of BMP-2 recycling remains unknown and requires further research.

For BMPRs, previous studies demonstrate that BMPRII and BMPRIa are regulated by Dullard, which is a phosphatase, upon endocytosis. When Dullard is inhibited, BMP signaling is enhanced, and when Dullard is active, BMPRIa and BMPRII are deactivated and dephosphorylated to inhibit BMP signaling [150,216,217]. Additionally, once the BMPRs are endocytosed, a ubiquitin ligase named Smurf1 was also identified to ubiquitinate the BMPR complexes and cause degradation [218,219,220,221]. Together, these proteins regulate BMP-2/BMPR activity intracellularly. 

## 3. Clinical Applications and Limitations of BMP-2

In 2002, the FDA approved clinical use of rhBMP-2, and it remains the only commercially available therapeutic that serves as an alternative to bone grafts [223]. Additionally, rhBMP-2 is available as a therapeutic in anterior lumbar interbody fusion during spinal fusions within tapered cages [49,50,51]. rhBMP-2 has also been used during cranioplasty, especially following maxillofacial injury, to induce osteogenesis [224]. Because of the promising and well-known osteogenic potential of rhBMP-2, this therapeutic also seems ideal for osteoporotic and osteoarthritic patients. In addition, it has been postulated that BMP-2 may be used to treat disorders such as multiple sclerosis, cardiovascular disease, anemia, atherosclerosis, renal calcification, and kidney failure [121,134,197,225,226,227,228,229,230]. However, over the past decade, several complications and side-effects have been identified after using rhBMP-2, potentially indicating that it is unsafe for bone fractures and other diseases [223]. 

Because BMP-2 is a vital and critical growth factor and holds clinical applications, it became a popular protein studied in the clinical research field. Additionally, studies demonstrated that BMPs themselves have been implicated in both tumor progression and suppression. BMP-2 specifically has been implicated in suppressing tumor development in human colorectal cancer cells [231]. However, it has also been implicated in tumor progression through stimulating epithelial to mesenchymal transition (EMT) and breast cancer stemness through Rb and CD44 [164]. In fact, low levels of BMP-2 have been implicated in poor patient prognosis in prostate cancer [232]. Due to these varying reports, it is suggested that use of BMP-2 in the clinic in cancer patients should be carefully considered [233].

BMP-2 was introduced into the clinic because of complications surrounding complex bone healing fractures, or delayed bone healing. At the time rhBMP-2 delivery, known as INFUSE (Infuse bone graft, lumbar tapered device), was with a bovine collagen matrix soaked in BMP-2 [223]. As stated previously, INFUSE has been associated with a variety of negative effects, which greatly limits its use in the clinic. This could be due to the amount of BMP-2 used for these procedures, especially in spinal fusion. Normally, there are 2 mg of BMP-2 in circulation and use in the human body. INFUSE uses 40mg of BMP-2 in order to promote its positive osteogenic effects [224,225]. However, the amount of BMP-2 used causes life-threatening complications through swelling in the neck. It was also discovered that only 75 ug of BMP-2 remained bound to collagen within the tapered cages, while the remaining amounts of BMP-2 precipitated onto the bovine cages, thus causing significant inflammation, swelling, and heterotrophic ossification [226,227]. Further elaborating on this administering technique, it has been demonstrated that Noggin is upregulated after BMP-2 is injected [234,235].This poses serious complications because Noggin negatively regulates BMP-2 and can prevent BMP-2’s course of action. Therefore, high concentrations of BMP-2 are needed to overcompensate, which leads to a variety of complications and side-effects. Additionally, therapeutics targeting Noggin has been ineffective, as Noggin can be resistant or insensitive to these treatments [236].

It has been hypothesized that the supraphysiological amounts of BMP-2 used in INFUSE cause the major adverse reactions. Therefore, limiting the amount of BMP-2 or creating slow-release vehicle delivery systems is of active research interest. There are several types of rhBMP-2 delivery systems: binding BMP-2 to a molecular vehicle through intermolecular forces, encapsulating or absorbed BMP-2, coupling by affinity interactions, non-site directed covalent coupling, or site-directed covalent coupling [237]. Many research groups prefer the non-covalent coupling of BMP-2 to a delivery vehicle, as covalent attachment of BMP-2 could affect its overall activity and later associations. INFUSE used collagen sponges, which are the most widely studied and characterized rhBMP-2 delivery systems. However, a major disadvantage to this medium is that it relies on an early burst-release of BMP-2 [238]. Other labs are working on other types of delivery vehicles, in order to provide a slow release system for BMP-2 within the body [237]. 

Recent data have shown that cells extracted from patients diagnosed with osteoporosis do not respond to BMP-2 stimulation, whereas cells extracted from patients diagnosed with osteoarthritis are still responsive [179]. This demonstrates that BMP-2 cannot enhance bone mineral density in patients diagnosed with osteoporosis, but still causes adverse side-effects such as radiculitis, hematoma, and an increased rate of microfractures [48]. As a result, BMP-2 may not be useful for treating osteoporosis, and alternative therapeutics are desperately needed (described in the next section).

In addition to osteoporotic patients’ unresponsiveness to BMP-2, rhBMP-2 injections also lead to an increased rate of osteolysis and microfractures [48]. Recently, clinical evidence demonstrated that after lumbar spinal fusion with rhBMP-2 injection in titanium tapered cages, at least three or more patients were diagnosed with osteolysis several months later [55,56,239]. Along with an increased bone resorption rate, an increased occurrence of radiculitis and nerve injury was also present after usage of rhBMP-2 [48,53,240,241,242]. Another major side-effect of using rhBMP-2 is ectopic bone formation, due to BMP-2 leaking out of the implant site. In fact, 70.1% of patients administered rhBMP-2 showed ectopic bone formation on a CT scan [223,243]. The quality of bone formed after rhBMP-2 use comes into question, as an increased risk of bone cyst formation was also observed. BMP-2 itself is an important growth factor for adipocytes (fat cells) in addition to osteoblasts; therefore when rhBMP-2 is administered, MSCs can be terminally differentiated into either osteoblasts or adipocytes, causing more lipid formation within the newly formed bone. While bone volume may be increased with the use of rhBMP-2, trabecular spacing is decreased and lipid deposition is increased, decreasing the overall quality of the bone [244,245]. Further, retrograde ejaculation was a common diagnosis after patients were injected with rhBMP-2 [246,247]. A significant black box warning was issued by the FDA in 2008 regarding the use of rhBMP-2, stating that there had been an increase in local inflammation responses causing cervical spinal swelling and death [248]. Therefore, with all these adverse complications, rhBMP-2 may not be suitable as a bone therapeutic. Current research is attempting to develop fluorescent BMP-2 analogs to elucidate the lack of responsiveness to rhBMP-2 [249,250].

### Alternative Therapeutics to BMP-2

Currently, osteoporosis and lone bone mineral density affects 54 million Americans (NOF, 2020). A commonly used treatment is the Human Parathyroid Hormone (Teriparatide [PTH 1-30]) therapy, which helps to control calcium homeostasis and has increased bone mineral density and trabecular bone mass [251,252,253]. Teriparatide is a peptide hormone that targets G-Protein-Coupled-Receptors and stimulates an increase of adenylate cyclase, which results in an increase in calcium that is then used to promote bone growth [254]. Teriparatide is classified as an anabolic drug and is intermittently injected subcutaneously [255]. When a patient stops receiving Teriparatide injections, bone mineral density decreases. However, bisphosphonates can then be provided to restore bone loss by inhibiting the activity of osteoclasts, which helps to preserve the newly formed bone [256,257,258]. Bisphosphonates can also be used without Teriparatide, as they are analogs of pyrophosphates and bind to hydroxyapatite to prevent bone resorption [257]. Use of these treatments can lead to hypercalcemia, increased risk of developing tumors, and kidney disorders [259]. Although Teriparatide and bisphosphonates increase bone mineral density and limit osteoclast activity, patients are still at risk of developing future complications. 

In addition, there are other current treatments that also target osteoclast activity and serve as antiresorptive drugs. For example, hormone replacement therapy, such as Selective Estrogen Receptor Modulator (SERM), was developed for post-menopausal women. Although this therapeutic replaces decreased estrogen, several side-effects have been noted. These include hot flashes, cramps, and a variety of thromboses and embolisms [252,260,261,262,263,264]. Another treatment is Denosumab, which binds to RANK-L to prevent activation of osteoclasts. Although this therapeutic has successfully decreased osteoclast activity and prevented fractures, it has led to side-effects, such as hypocalcemia, jaw osteonecrosis, and tumor progression [265,266,267]. 

Romosozumab was recently approved as an osteoporotic treatment in 2019. This drug has demonstrated a decrease in bone resorption while increasing bone formation [268]. It functions by binding directly to SOST, which is a BMP-antagonist that prevents activation of Smad-signaling by binding to the low-density lipoprotein-related protein (LRP5) receptor, thereby preventing activation of the β-Catenin/Wnt/BMP-signaling pathways and osteogenic genes [204,269]. To date, Romosozumab has decreased the occurrence of spinal injuries, but there have been two reported cases of jaw osteonecrosis and one case of hepatitis [268,270]. Due to the novelty of this drug, other benefits and side-effects remain unknown. 

A novel peptide named Casen Kinase 2.3 (CK2.3) was manufactured and developed by our lab to specifically bind CK2 and prevent its association with BMPRIa. With CK2.3, the BMP-signaling pathway is activated without exogenous BMP-2 by blocking CK2 binding and activating downstream signaling pathways. CK2.3 has successfully activated downstream effector proteins, such as Smad1/5/8, pERK, and Akt [31]. Further, CK2.3 is able to enter the cell, primarily through caveolae, indicating that it is biologically active [271]. The biological activity of CK2.3 was also demonstrated as the peptide induced trabecular bone formation and bone mineralization while limiting osteoclastogenesis, indicating its potential use as an osteoporotic therapeutic [31,179,182,183]. However, the trafficking of CK2.3 and its precise course of action within osteoblastogenesis and osteoclastogenesis are still unknown and are current research topics. Another CK2 inhibitor, namely CX-4945, inhibits CK2/Akt signaling and decreases osteoclast formation and activity [272,273,274]. CX-4945 is currently in clinical trial phases and requires further testing before being administered as a treatment. The current available treatments stated above along with their additional side-effects are summarized in Table 3. 

Another important area of research is the usage of BMP-2 mimetic peptides to induce bone formation and Smad signaling. These peptides are of importance because they currently exert osteoinductive properties but are not commercially available therapeutics. One of these peptides is P17-BMP-2. P17-BMP-2 enhanced bone repair after injury, as well as stimulating an increase in osteoblast differentiation and bone regeneration [275,276]. Additionally, other mimetic peptides including P20 and P24 have demonstrated osteogenic properties and the ability to differentiate MSCs into osteoblasts; thus, these peptides have the potential to be used in bone repair and/or regeneration, but require further study and experimentation [275,276,277,278,279]. 

## 4. Conclusions

BMP-2 is a powerful player in all organs. Potential treatments for osteoporosis, osteopenia, osteoarthritis, and coronary artery disease with BMP-2 and/or its signaling pathways are in development. However, to date, the FDA has only approved BMP-2 during anterior lumbar interbody spinal fusions, cranioplasties, and maxillary facial reconstructive surgery. Therefore, the bone and skeletal disorders were the main focus of this review.

**Figure 4:**
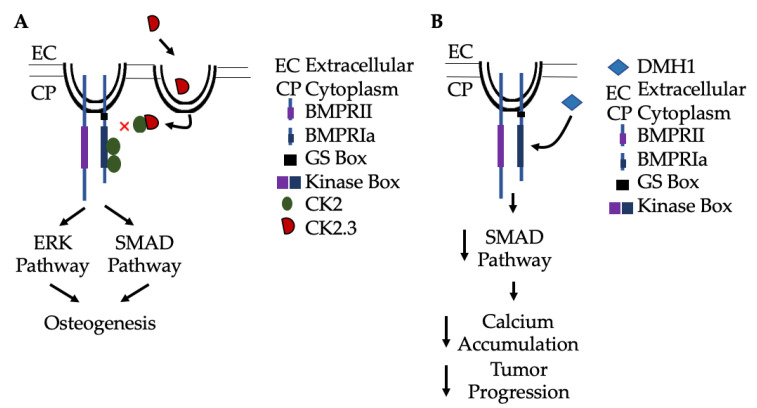
Activation and inactivation of Smad signaling without exogenous BMP-2. (**A**) CK2.3 is endocytosed into cells via caveolae, and then binds to CK2 to prevent its association with BMPRIa. This results in activation of Smad and non-Smad signaling pathways (such as ERK), leading to osteogenesis. (**B**) Alternatively, BMPRIa inhibitors, such as Dorsomorphin (DMH1), specifically bind to the receptor and inhibit Smad-signaling to limit arterial calcification by limiting Ca^2+^ accumulation. Further, DMH1 has been implicated in slowing tumor progression and metastasis.

During development, BMP-2 promotes cardiogenesis, neurogenesis, development of the ocular system, and development of the D/V and A/P axes. BMP-2 deficiency during development ultimately leads to embryonic or post-natal lethality due to its diverse functions. In addition, over-expression of BMP-2 can lead to myopia or neural tube closure defects. Therefore, its activity is tightly regulated throughout embryogenesis, development, and adulthood. Further into adulthood, BMP-2 is responsible for inducing endochondral and intramembranous ossification, activation of the adipogenic and chondrogenic signaling pathways, and maintenance of neurons, lungs, and cardiac tissue. Because of its versatile functions, BMP-2 has been used as an osteoinductive agent. However, evidence during the past decade has demonstrated adverse side-effects of BMP-2, such as hematoma formation, osteolysis, increased spontaneous fractures, coronary artery disease, myopia, and other complications. Other methods to induce osteogenesis, such as PTH therapies, bisphosphonates, and Romosozumab have been implemented, but they also pose side-effects, such as tumor growth, hypercalcemia, and jaw osteonecrosis. Synthesized peptides, such as Romosozumab and CK2.3, promote osteoblastogenesis and limit osteoclastogenesis, making them possible therapeutics for osteoporosis by BMP-signaling without exogenous BMP-2 (Figure 4). Additionally, other small peptides, such as Dorsomorphin (DMH1), can target BMPRIa to inhibit BMP-signaling. Inhibition of this pathway leads to decreased calcium release, reduced medial arterial calcification, and decreased metastasis in breast cancer [306,307,308]. With these applications, the BMP-signaling pathway can be implicated to treat a variety of diseases. However, the complete functions and side-effects of these peptides are still unknown. With millions of Americans diagnosed with osteoporosis, cardiovascular disease, and cancer, there is a desperate need for novel therapeutics that can utilize the BMP-signaling pathway to treat these disorders with no adverse side-effects. 

## Figures and Tables

**Table 1:** Known agonists and antagonists of BMP-2.

Protein/Gene	Type of Protein	Function	References
Brorin	Glycoprotein	Inhibits BMP-2 activity	[184,185,186]
Cerberus	Cytokine	Blocks BMP-2 signaling	[187,188]
Chordin	Glycoprotein	Binds directly to BMP-2 to prevent its activity	[170,171,177,189,190]
DAN Family	Glycoproteins	Binds directly to BMP-2 and inhibits its activity	[191]
Follistatin	Glycoprotein	Binds directly to BMP-2 to prevent its activity	[173,192,193,194]
FLRG	Glycoprotein	Downregulates BMP-2 and limits its activity	[194,195,196]
Grem2	Glycoprotein	Inhibits BMP-2 activity	[197,198]
Noggin	Glycoprotein	Binds directly to BMP-2 to prevent its activity	[9,172,199,200]
Sclerostin (SOST)	Glycoprotein	Binds to LRP5/6 to prevent Wnt and BMP-2 signaling	[201,202,203,204]
*Shrew*	Shrew-1: Transmembrane protein	Enhances peak BMP-2 signaling activity	[169,170]
*Twisted Gastrulation*	Produces small cysteine rich protein	Can enhance or inhibit BMP-2 activity	[169,205,206,207,208,209,210]
USAG-1	Glycoprotein	Physically interacts with BMP-2 to prevent signaling	[211,212]

**Table 2:** Known proteins associated with BMPRs.

Protein	References
FGFR substrate	[222]
PKCβ	[222]
Rab geranylgeranyl transferase	[222]
MOS	[222]
MAPKKK8	[222]
CtBP	[222]
Forkhead L1 TF	[222]
LIM hd 1	[222]
p50b	[222]
SemF	[222]
hnRNP R	[222]
Neurobeachin	[222]
Tubulin β5	[222]
Onconin-90	[222]
ARP8	[222]
Arylsulfotransferase	[222]
Carboxylesterase 3	[222]
Contrapsin	[222]
Protein tyrosine kinase 9	[222]
C4b-binding protein	[222]

**Table 3:** Current osteoporosis treatments, function of the drugs/treatments, and the drug/treatment side-effects.

Drug/Treatment	Drug/Treatment Function	Side-Effects of Drug/Treatment	References
Bisphosphonates	Antiresorptive	Gastrointestinal discomfort; hypocalcemia; esophageal cancer; jaw osteonecrosis; decreased bone turnover rate	[252,253,257,258,259,280,281,282,283,284,285,286,287,288,289,290,291,292,293,294]
Calcitonin	Antiresorptive	Nausea; increased risk for cancer; allergic reactions; hypocalcemia	[41,295]
Denosumab	Antiresorptive	Tumor progression; hypocalcemia; jaw osteonecrosis; pancreatitis	[265,266,267]
Hormone replacement therapy (i.e., selective estrogen receptor modulator [SERM])	Antiresorptive	Hot flashes; leg cramps; pulmonary embolism; deep vein thrombosis; retinal vein thrombosis; thromboembolism; invasive breast cancer	[260,261,262,263,264,296]
Romosozumab	Antiresorptive and Anabolic	Jaw osteonecrosis; hepatitis; nasopharyngitis; hypercalcemia; arthralgia	[268,270,297,298,299,300]
Teriparatide (PTH 1–34)	Anabolic	Nausea; vomiting; headaches; hypercalcemia; hypercalciuria; hypomagnesemia	[252,253,301,302,303,304,305]
